# Comment on “Apical Sparing of Longitudinal Strain and Myocardial Fibrosis in Hypertensive Patients and Spontaneously Hypertensive Rats: Based on Speckle Tracking and Histological Analysis”

**DOI:** 10.1002/clc.70384

**Published:** 2026-06-15

**Authors:** Hussnain Bilal

**Affiliations:** ^1^ Department of Medicine Allama Iqbal Medical College Lahore Punjab Pakistan

To the Editor,

I read with great interest the recent article by Huang et al. exploring the phenomenon of apical sparing of longitudinal strain and myocardial fibrosis in hypertensive patients and spontaneously hypertensive rats. The combined use of speckle tracking echocardiography and histological validation is commendable and adds mechanistic depth to the understanding of regional myocardial heterogeneity in hypertensive heart disease. Nonetheless, several methodological and interpretative aspects merit further discussion to strengthen the clinical applicability of the findings [1].

First, the interpretation of the proportion apical strain index P ap deserves caution. As defined, P ap is mathematically dependent on basal and midventricular strain values. Therefore, an increase in P ap may predominantly reflect deterioration of basal or mid‐segment strain rather than true preservation or compensatory enhancement of apical mechanics. This is particularly evident as LS ap still declined in the hypertensive LVH group despite a higher P ap. Similar concerns regarding ratio‐based strain indices have been raised in other disease states where relative apical sparing may amplify basal dysfunction rather than represent an independent physiological marker [2].

Second, the correlation analyses linking P ap with indices of hypertrophy and diastolic dysfunction were limited to unadjusted bivariate testing. Given the close interrelationship between LV mass wall thickness and strain parameters, it remains uncertain whether P ap provides incremental information beyond established markers such as LVMI or GLS. Multivariable modeling, adjusting for blood pressure severity, age, and LV geometry, would have clarified whether P ap is independently associated with remodeling or merely reflects collinearity among structural parameters. Prior work in hypertensive cohorts has highlighted the importance of such adjustment when proposing novel deformation indices [3].

Third, the mechanistic link between strain heterogeneity and myocardial fibrosis in humans remains indirect. While the SHR model elegantly demonstrated basal predominant collagen deposition, no myocardial tissue characterization was performed in the clinical cohort. Without cardiac magnetic resonance or other fibrosis‐sensitive imaging modalities, extrapolating animal histological patterns to human hypertension remains speculative. Previous studies have shown that diffuse and focal fibrosis assessed noninvasively may only partially align with deformation abnormalities, underscoring the need for direct human validation [4].

Finally, the translational generalizability of the animal findings warrants careful consideration. SHR represents a model of severe, longstanding hypertension with blood pressure levels substantially exceeding those typically observed in early human disease. The absence of significant GLS reduction in SHR despite marked fibrosis further complicates direct comparison with the clinical cohort. Differences in ventricular geometry, loading conditions, and segmentation between rodent and human hearts may partly account for these discrepancies, as previously discussed in comparative myocardial mechanics research [5].

In conclusion, this study provides valuable insight into regional myocardial remodeling in hypertension. Addressing these methodological considerations in future prospective and multimodality studies would help clarify the independent clinical value of apical sparing indices and strengthen their role as potential biomarkers of hypertensive heart disease progression.

## Funding

The author has nothing to report.

## Data Availability

Data sharing is not applicable to this article as no data sets were generated or analyzed during the current study.
